# The mechanistic target for rapamycin pathway is related to the phosphorylation score for estrogen receptor-α in human breast tumors *in vivo*

**DOI:** 10.1186/bcr3660

**Published:** 2014-05-22

**Authors:** Anuraag Shrivastav, Mary Christine Bruce, Danira Jaksic, Tarek Bader, Srinivas Seekallu, Carla Penner, Zoann Nugent, Peter Watson, Leigh Murphy

**Affiliations:** 1 Department of Biochemistry and Medical Genetics and the Manitoba Institute of Cell Biology, University of Manitoba and CancerCare Manitoba, 675 McDermot Avenue, Winnipeg, MB R3E 0V9, Canada; 2 Department of Biology, University of Winnipeg, Winnipeg, MB, Canada; 3 Manitoba Breast Tumour Bank, Manitoba Institute of Cell Biology, University of Manitoba and CancerCare Manitoba, Winnipeg, MB, Canada; 4 Tumour Tissue Repository and Deeley Research Center, BC Cancer Agency, Victoria, BC, Canada

## Abstract

**Introduction:**

A phosphorylation score for estrogen receptor-alpha (ERα), called P7 score, was shown previously to be an independent prognostic factor in breast cancer patients treated with tamoxifen. Since mechanistic target of rapamycin (mTOR) activation is implicated in resistance to endocrine therapy in breast cancer we determined whether mechanistic target of rapamycin complex 1 (mTORC1) activation, measured by phosphorylation on S2448 (p-mTOR), was associated with the P7-score and/or clinical outcome in the same cohort.

**Methods:**

mTOR phosphorylation status was determined at S2448 residue *in vivo* by immunohistochemistry in a cohort of more than 400 well-characterized ERα positive breast tumors. MCF7 cells were treated with estrogen and activation of mTOR pathway was determined by Western blotting.

**Results:**

Contrary to earlier reports, p-mTOR expression, measured by immunohistochemistry, was negatively associated with size and nodal status. Additionally, p-S2448 mTOR expression was positively correlated with p-S118- ERα, p-S167-ERα and p-S282-ERα but negatively correlated with p-T311- ERα. Consistent with these, p-S2448 mTOR was negatively associated with P7-score and was significantly associated with overall survival (OS) (hazard ratio (HR) = 0.61, *P* = 0.028, 95% confidence interval (CI) 0.39 to 0.95, *n* = 337) and relapse-free survival (HR = 0.58, *P* = 0.0032, 95% CI 0.41 to 0.83, *n* = 337) following univariate but not multivariate analysis. Furthermore, we show that estrogen can regulate phosphorylation of mTOR and its down stream target p70S6 kinase. Additionally, recombinant mTOR can phosphorylate ERα *in vitro*.

**Conclusions:**

These data suggest that in breast tumors where there is intact estrogen regulated signaling, mTOR is regulated by estrogen and therefore associated with an increased likelihood of responsiveness to endocrine therapy.

## Introduction

The estrogen receptor-α (ERα) status of breast tumors is the gold-standard marker for predicting response to endocrine therapy. This is due primarily to its central role in estrogen signaling within ERα + breast cancer [1]. However, ERα status as currently measured does not accurately predict treatment response since at least 50% of ERα + tumors are *de novo* resistant to endocrine therapies such as tamoxifen, and many of those initially sensitive will acquire resistance despite the continued expression of non-mutated ERα in most cases [2]. ERα, like many other proteins, can be post-translationally modified [3]. Post-translational modifications (PTMs), such as phosphorylation, acetylation, methylation and ubiquitination of ERα, have been identified and, in some cases, shown to affect ERα activity [3]. Investigation of the relevance of phosphorylated forms of ERα *in vivo* in human breast tumors revealed that many breast tumor biopsy samples have detectable phosphorylated ERα [4,5]. Recently, we determined expression of seven different phosphorylated residues on ERα in breast cancer samples from patients who subsequently were treated with tamoxifen, and found that multiple tumors expressed combinations of phospho-ERα epitopes [6]. We also established that detection of some of these phosphorylated sites was significantly associated with good and others with poor clinical outcome [6,7]. This led us to define an ERα phosphorylation score which takes into account the presence of all seven phosphorylated ERα epitopes detected in any one tumor. This so-called P7-score was found to be significantly associated with overall survival from breast cancer death and relapse free survival (RFS) in multivariate analysis [6]. Such data support the hypothesis that a phosphorylation code for ERα exists that is a more accurate prognostic and, possibly, treatment response marker than determination of the expression of ERα alone. It also suggests that ERα is a central node at which integration of diverse signals occurs to regulate breast cancer growth and survival. We have hypothesized that the P7-score represents the balance of estrogen-dependent (ligand-dependent) and ligand-independent ERα signaling associated with any tumor [6]. These data highlight the potential role played by kinases in breast tumors *in vivo*[8-10] responsible for maintaining the ERα phosphorylation code, as they may provide targets for development of new ‘endocrine’ or alternative therapies.

It has been suggested that increased activation of the mechanistic target of rapamycin (mTOR) pathway [11], possibly through the PI3K/Akt pathways, plays a role in endocrine resistance exhibited by some ERα + breast cancer cells, since inhibition of mTOR signaling with rapamycin could restore sensitivity to tamoxifen in laboratory models of resistance [12,13]. Furthermore, p70-S6kinase (p70S6K), a downstream target of activated mTOR, was shown to directly phosphorylate ERα on Ser167 and increase the transcriptional activity of ERα [14]. The possibility exists that increased activated mTOR may help drive ligand-independent ERα signaling and short circuit the ligand-dependent pathway that is most sensitive to inhibition by endocrine therapies. In this study, we have evaluated the relationship between activated mTOR signaling and the ERα phosphorylation score, as a measure of the balance of ligand-dependent and -independent ERα signaling, using human breast cancer cases, where the patient subsequently received adjuvant tamoxifen therapy.

## Methods

### Materials/reagents

Recombinant human ER (rh-ERα) was from Invitrogen (Carlsbad, CA, USA), recombinant human mTOR (rh-mTOR, catalytic subunit) was from BPS Bioscience (San Diego, CA, USA) and recombinant human p70S6 kinase (rh-p70S6K) was from R&D Systems, Inc (Minneapolis, MN, USA). AZD8055, a selective, ATP-competitive mTOR kinase inhibitor was from Cedarlane (Burlington, ON, Canada). PF-4708671, a selective p70-S6kinase inhibitor, was from EMD Millipore Co. (Cedarlane, Burlington, ON, Canada).

### Tissue microarrays

All primary invasive breast cancers used in this study were from the Manitoba Breast Tumor Bank (MBTB, CancerCare Manitoba and University of Manitoba) [15,16]. MBTB embraces the policies and operating protocols of the Canadian Tumor Repository Network [17] and operates with approval from the Research Ethics Board of the Faculty of Medicine, University of Manitoba. The histopathology of MBTB biospecimens was previously assessed and entered into a computerized database to enable selection based on tissue composition and clinical-pathological parameters. Tissue collection and sample selection for tissue microarray (TMA) construction was reported before [6]. ERα positive status was determined by ligand binding assay (>3 fmol/mg protein) at the time of diagnosis and confirmed by immunohistochemistry (IHC) in the TMAs as previously described [5]. Although 450 cases were represented on the original TMAs, due to exhaustion of some tumor cores from previous use of the TMAs, the tumor numbers (n) analyzed for some markers were less than 450. The study cohort characteristics have been previously published [6] and did not change significantly due to exhausted tumor core drop out: the current cohort characteristics are progesterone receptor (PR)-positive (>20 fmol/mg protein), 62.5% (261/336); PR-negative, 37.5% (126/336); low-grade, 27.7% (93/336); intermediate-grade, 61.6% (207/336); high-grade,10.7% (36/336); tumor size <2.5 cm, 55.5% (187/337); tumor size ≥2.5 cm, 44.5% (150/337); age <50 years, 6.9% (23/335); age >50 years, 93.1% (312/335); node-negative, 49.6% (164/331); node-positive, 50.5% (167/331). The median follow-up was 99 months (range 9 to 217 months).

### Antibodies

The P7-scores for the study cohort were previously determined and reported [6]. The antibodies used for IHC were validated as previously described [5]: mTOR (rabbit monoclonal, 7C10) and mTOR phosphorylated on serine 2448 (p-S2448 mTOR; rabbit monoclonal, 49 F9), antibodies and blocking peptides were from Cell Signaling Technology Inc. (NEB Ltd, Whitby, ON, Canada). p70S6kinase (p70S6K, N-terminal; rabbit monoclonal, clone E343, cat # 1494–1), p70S6K phosphorylated on threonine 389 (p-T389 p70S6K; rabbit monoclonal, clone E175, cat # 1175–1, or Cell Signalling, rabbit monoclonal 108D2, cat#9234) and blocking peptides were from Epitomics Inc (Burlingame, CA, USA). Validation of p-2448 mTOR and p-T389p70S6K is shown in Additional file 1: Figure S1. Similar validations were undertaken for p70S6K and mTOR (not shown). The anti-phosphoserine antibody (ab9332) was from Abcam Inc (Cambridge, MA, USA). The antibodies used for immunoprecipitation/Western blotting were against total mTOR/FKBP12-rapamycin complex-associated protein (FRAP) (sc-1549-R) and total ERα (sc-543) from Santa Cruz Biotechnology Inc. (Santa Cruz, CA, USA). Although in Additional file 1: Figure S1 there appears to be a partial reduction in IHC signal due to the non-phosphorylated peptide (+mTOR) and an irrelevant p-peptide (+pS118), close examination of Additional file 1: Figure S1C and 1D and comparison to Additional file 1: Figure S1A shows that the dynamic signal intensity range among positive cells is similar. In Additional file 1: Figure S1D there is a very dark brown staining section (going clockwise from the top of the circle) between 180° and 270° as well as a small circle of intense staining at approximately 180°; in Additional file 1: Figure S1C an intensely staining focus of cells is found at approximately 90° and 135°. These are similar in intensity to regions in Additional file 1: Figure S1A. Even in Additional file 1: Figure S1A there is a range of staining intensities reflecting the heterogeneous nature of tumors and the cells within them. Each different staining is done on a serial section which increases the heterogeneity. It should be noted, as well, that the counter-stain in Additional file 1: Figure S1A is generally more intense than Additional file 1: Figure S1C and 1D, which affects the perception of intensity. In contrast, all signal is lost in Additional file 1: Figure S1B where the antibody is pre-absorbed with excess of the specific phospho-peptide. The data seen in these figures support the well-known heterogeneity of expression of any protein that seems to occur in breast tumor cells *in vivo* in a breast biopsy specimen. So perceived differences, we argue, are due primarily to tissue composition and tumor cell heterogeneity and not due to a lack of phospho-epitope specificity, although we cannot completely eliminate this possibility.

### Tissue collection times

As previously described [5], a cohort of breast tumors for which the collection time has been defined previously [5], is available in the Manitoba Tumor Bank. This timed collection cohort was used to determine if detection of p-mTOR and p-p70S6K varied significantly with time of biospecimen collection. Formalin fixed-paraffin embedded blocks from 133 cases had sufficient material to be used for this study. IHC for both p-mTOR and p-p70S6K was carried out on adjacent sections. Within this cohort the collection time ranged from 5 to 276 minutes (mean 56 minutes and median 45 minutes). Although there may be a trend for the p-mTOR IHC score to decrease with time no statistically significant relationship between collection time and p-mTOR (spearman *r* = −0.16, *P* = 0.066, n = 133) or p-p70S6K (spearman *r* = 0.064, *P* = 0.47, n =130) was found. The tumors were also divided into groups based on collection times of ≤30 minutes versus >30 minutes. Mann–Whitney two tailed analyses showed no significant differences in the IHC score between the two time groups for either p-mTOR (*P* = 0.064) or p-p70S6K (*P* = 0.81) as illustrated in Additional file 2: Figure S2.

### Immunohistochemistry

IHC for TMAs was performed as described previously [18]. Serial sections (approximately 5 μm) were stained with antibodies as previously described [5]. Briefly, sections were submitted to antigen retrieval (CC1, Ventana Medical Systems, Tucson, AZ, USA) using an auto-immunostainer (Discovery Staining Module, Ventana Medical Systems), followed by one-hour incubation with primary antibody and 32-minute incubation with secondary antibody. Primary antibody concentrations initially applied to the Ventana instrument were 1:50 for antibodies to p-S2448 mTOR, mTOR, p70S6K and p-T389 p70S6K translating into final concentrations of 1:150 after 1:3 dilution with buffer dispensed onto the slide with the primary antibody.

### Quantification and cut-off selection

Slides were scored using standard light microscopy. IHC scores were derived from assessment of both average staining intensity across the two tumor cores (scale 0 to 3) and percentage of positive cells (0 to 100%). These two scores, when multiplied, generate an IHC or H-score of 0 to 300. Cytoplasmic staining for mTOR and p-S2448 mTOR was scored. Little nuclear staining of mTOR or p-S2448 mTOR was seen in this study cohort. Cytoplasmic and nuclear staining for p70S6K and p-T389 p70S6K were scored. TMAs were evaluated independently by two investigators (AS, CP). Where discordance was found, cases were re-evaluated to reach consensus. Since no relevant clinical cut-off points are presently reported for mTOR, p-S2448 mTOR, p70S6K and p-T389 p70S6K, positivity reported in this study was empirically based on IHC scores greater than the 50th percentile. RFS was defined as time to first recurrence or death due to breast cancer (censors were other death) and overall survival (OS) was defined as time to death due to breast cancer (censors were other death).

### Cell culture and immunoprecipitation

MCF7, ER + human breast cancer cells were routinely cultured in (D)MEM containing 5% (v/v) fetal bovine serum (FBS), 1% (w/v) glucose, glutamine and penicillin–streptomycin (5% complete medium (CM)). For experiments, cells were estrogen depleted and serum starved for four days in serum free-phenol red-free (D)MEM before treatment with estradiol-17β (10 nM) or vehicle control (ethanol) for various times. Cells were harvested at the various time points and subjected to SDS polyacrylamide electrophoresis (SDS-PAGE) and Western blotting as previously described [19]. For immunoprecipitation, cells were grown as described above, washed twice with cold PBS and treated with 2 mM dithiobis(succinimidylpropionate) (DSP) for two hours at 4°C. The cross-linking was quenched with 20 mM Tris–HCl for five minutes at room temperature. Following that, cells were lysed by sonication in lysis buffer (10 mM Hepes pH 7.5, 10% glycerol, 150 mM NaCl, 1% NP40, 0.5% sodium deoxycholate, 0.1% SDS) supplemented with complete protease inhibitor cocktail (Roche, Mississauga, ON, Canada), 1 mM phenyl methyl sulfonyl fluoride (PMSF), 1 mM Na3VO4, and 5 mM NaF. Lysates were incubated with antibody overnight on a rotator at 4°C. Antibody-bound protein complexes were precipitated from lysates using Dynabeads Protein G (Life Technologies, Burlington, ON, Canada). Dynabeads were washed six times with wash buffer (10 mM Hepes pH 7.5, 10% glycerol, 150 mM NaCl, 1% NP40), followed by addition of sample buffer (50 mM Tris–HCl pH6.8, 2% SDS, 6% glycerol, 0.1 M dithiothreitol (DTT)). Protein complexes were resolved by SDS-PAGE and Western blotting, as above.

### *In vitro* kinase assays

Recombinant proteins (300 ng rh-ERα, 100 ng rh-mTOR, 100 ng rh-p70S6K) were incubated alone or together in kinase buffer (5X kinase buffer is 25 mM MOPS, 12.5 mM β-glycerophosphate, 25 mM MgCl2, 5 mM EGTA, 2 mM EDTA, 0.25 mM DTT) with or without, a final concentration of 2 mM ATP, usually in a final volume of 25 μl. Incubation was for 30 minutes at 30°C; reactions were stopped by freezing. For inhibition assays, rh-ERα was pre-incubated with 100 nM AZD8055, 10 μM PF-4708671 or vehicle control (DMSO) for 15 minutes at 30°C, prior to the addition of ATP. Thereafter, reaction mixtures were thawed and subjected to SDS-PAGE electrophoresis and Western blot analysis.

### Statistical methodology

Survival analysis used Cox regression analyses to examine hazard ratios (HR). Each model was tested and all complied with the assumption of proportional hazard. These statistical analyses were performed using SAS™version 9.2. The probabilities shown in the single predictor models are not corrected for multiple comparisons. The probabilities in the multiple predictor model take into account the presence of the other predictors, that is, tumor size, nodal status, grade, PR expression, P7-score and p-mTOR.

## Results

### mTOR and pS2448-mTOR expression in human breast tumors

Previously constructed TMAs containing multiple samples of ERα + breast tumors were interrogated. IHC staining for both p-S2448 mTOR and total mTOR was observed and was primarily cytoplasmic; however, nuclear staining of both occurred in a minority of tumors (approximately10%).

Unexpectedly, it was found that total mTOR expression was negatively correlated with size and nodal status (Spearman correlation r = −0.206, *P* = 0.0002, n = 329; r = −0.27, *P* <0.0001, n = 323, respectively). Furthermore, p-S2448 mTOR was also found to be negatively associated with size and nodal status (r = −0.122, *P* = 0.026, n = 337; r = −0.15, *P* = 0.0068, n = 331, respectively). When tumors were divided into node negative and positive categories the median IHC scores for mTOR were significantly different (median IHC score 225 versus 180 respectively, *P* < 0.0001, Mann–Whitney two tailed, Additional file 3: Figure S3). Similarly, p-S2448 mTOR IHC scores were significantly different between node negative and positive tumors (median IHC score 90 and 70, *P* = 0.0073, respectively, Mann–Whitney two tailed, Additional file 3: Figure S3). When tumors were dichotomized into small (<2 cm) and large (≥2 cm) size, the median H scores for mTOR were significantly higher in small versus large tumor size (median H score 225 versus 180, respectively, Mann–Whitney two tailed *P* = 0.012, Additional file 4: Figure S4). In contrast, the same analysis for p-S2448 mTOR showed no significant difference (Additional file 4: Figure S4). Together these data suggested the possibility that an activated mTOR pathway, possibly associated with mTORC1, is a good prognostic factor in primary human ERα + breast cancer.

### Association of mTOR/p-S2448 mTOR with clinical outcome in patients treated with tamoxifen

**Figure 1:**
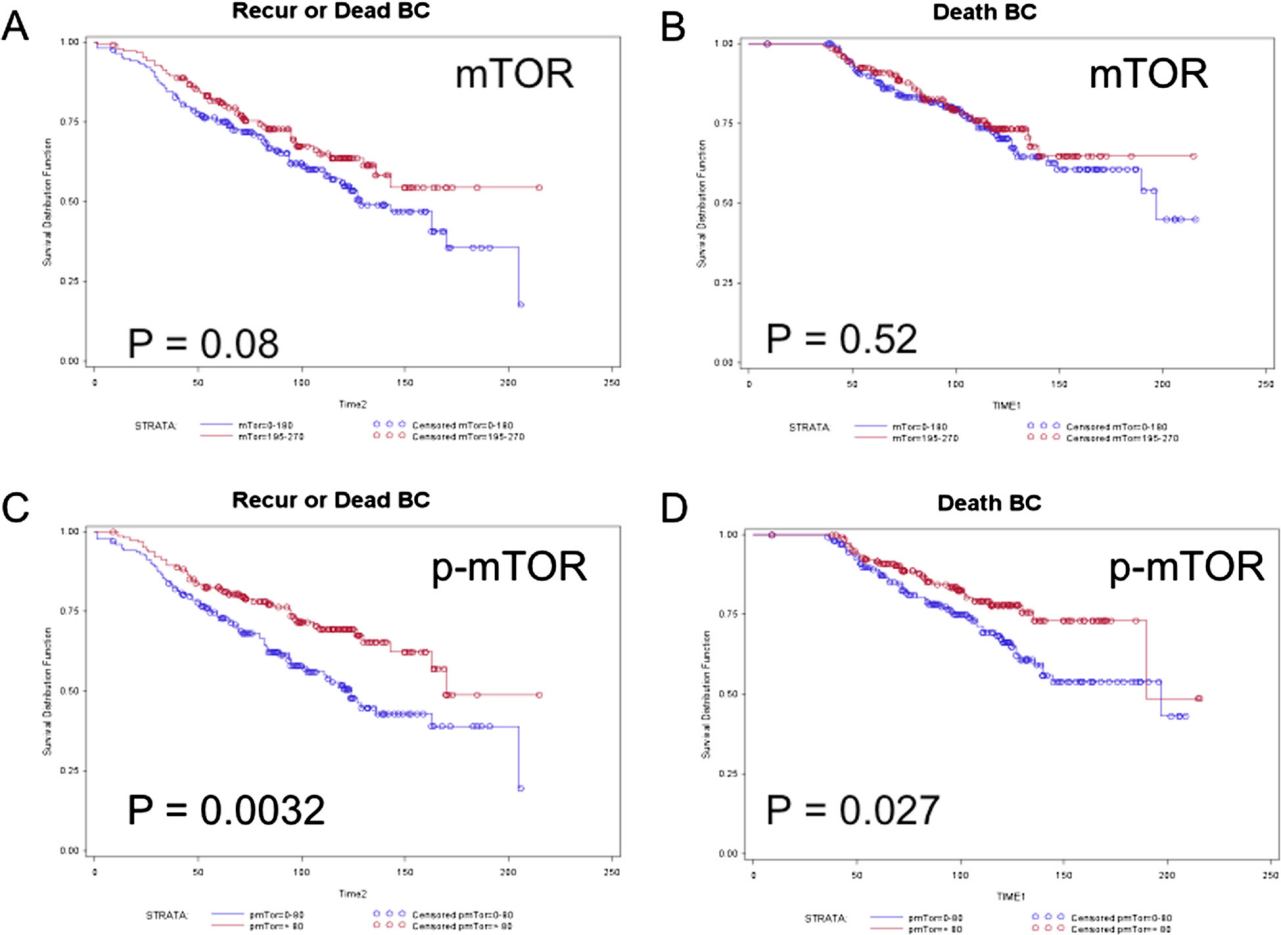
**Kaplan-Meier estimates of relapse free survival from breast cancer recurrence (A, C) or breast cancer specific death (B, D) with respect to expression of total mTOR (A, B) and p-mTOR (C, D). ***P* value represents the significance of a simple survival analysis without the proportional hazard assumption that was applied in the analyses presented in Tables 1 and 2. mTOR, mechanistic target of rapamycin.

The cohort of breast cancer cases interrogated for mTOR and p-S2448 mTOR represented primary ERα + tumors from patients who received adjuvant tamoxifen therapy after surgery. Therefore, the relationship of mTOR and p-S2448 mTOR to clinical outcome defined by RFS (RFS = endpoint recurrence and/or death due to BC, Table 1) and OS from death due to breast cancer (OS = endpoint death due to breast cancer, Table 2) was determined. Expression of mTOR was not significantly associated with clinical outcome, as shown in Table 1 and Figure 1A and B. However, high levels of p-S2448 mTOR (defined by > median H score 80) were found to be significantly associated with better clinical outcome, both OS (Table 2, HR = 0.61, *P* = 0.028, 95% CI 0.39 to 0.95, n = 337) and RFS (Table 1, HR = 0.58, *P* = 0.0032, 95% CI 0.41 to 0.83, n = 337) as shown in Figure 1C and D. However, this did not remain significant on multivariate analysis.

**Table 1:** Multivariate analysis of factors associated with recurrence free survival (RFS)

**Single predictor**	**Number**	**Hazard ratio**	**95% CI on HR**	***P***
Age 50+ versus <50	420	1.57	0.80 to 3.07	0.19
Tumor >2.5 cm	422	1.85	1.36 to 2.51	<.0001
Node Pos versus Neg	415	2.02	1.48 to 2.76	<.0001
Grade (Low, Mid High)	420	1.45	1.12 to 1.88	0.0055
PR_LBA >20	421	0.6	0.44 to 0.81	0.0010
P7 Score High	340	2.23	1.37 to 3.61	0.0012
pmTor >80	337	0.58	0.41 to 0.83	0.0032
mTor >180	329	0.73	0.51 to 1.04	0.084
**Multi predictor**				
Age 50+ versus <50	277	1.34	0.53 to 3.36	0.54
Tumor >2.5 cm		1.77	1.20 to 2.61	0.0041
Node Pos versus Neg		1.61	1.07 to 2.43	0.022
Grade (Low, Mid High)		1.21	0.88 to 1.64	0.24
PR_LBA >20		0.61	0.42 to 0.89	0.011
P7 Score High		1.77	0.99 to 3.18	0.054
pmTor >80		0.80	0.54 to 1.20	0.28

CI, confidence interval; mTOR, mechanistic target of rapamycin; PR_LBA, Progesterone Receptor Ligand Binding Assay.

**Table 2:** Multivariate analysis of factors associated with overall survival from death due to breast cancer (OS)

**Single predictor**	**Number**	**Hazard ratio**	**95% CI**	***P***
Age 50+ versus <50	420	1.67	0.74 to 3.81	0.22
Tumor >2.5 cm	422	2.27	1.56 to 3.30	<.0001
Node Pos versus Neg	415	2.06	1.42 to 3.00	0.0002
Grade (Low, Mid High)	420	1.38	1.02 to 1.87	0.035
PR_LBA >20	421	0.61	0.42 to 0.87	0.0068
P7 Score High	340	2.78	1.45 to 5.35	0.0022
pmTor >80	337	0.61	0.39 to 0.95	0.028
mTor >180	329	0.87	0.56 to 1.35	0.53
**Multi predictor/Selection model**				
Age 50+ versus <50	277	0.93	0.33 to 2.68	0.90
Tumor >2.5 cm		1.98	1.23 to 3.21	0.0053
Node Pos versus Neg		1.92	1.14 to 3.26	0.015
Grade (Low, Mid High)		1.21	0.83 to 1.76	0.32
PR_LBA >20		0.70	0.44 to 1.11	0.12
P7 Score High		2.57	1.09 to 6.06	0.032
pmTor >80		0.95	0.58 to 1.55	0.84

CI, confidence interval; mTOR, mechanistic target of rapamycin; PR_LBA, Progesterone Receptor Ligand Binding Assay.

### Association of mTOR/p-S2448 mTOR with the P7-phosphorylation score for ERα

**Figure 2:**
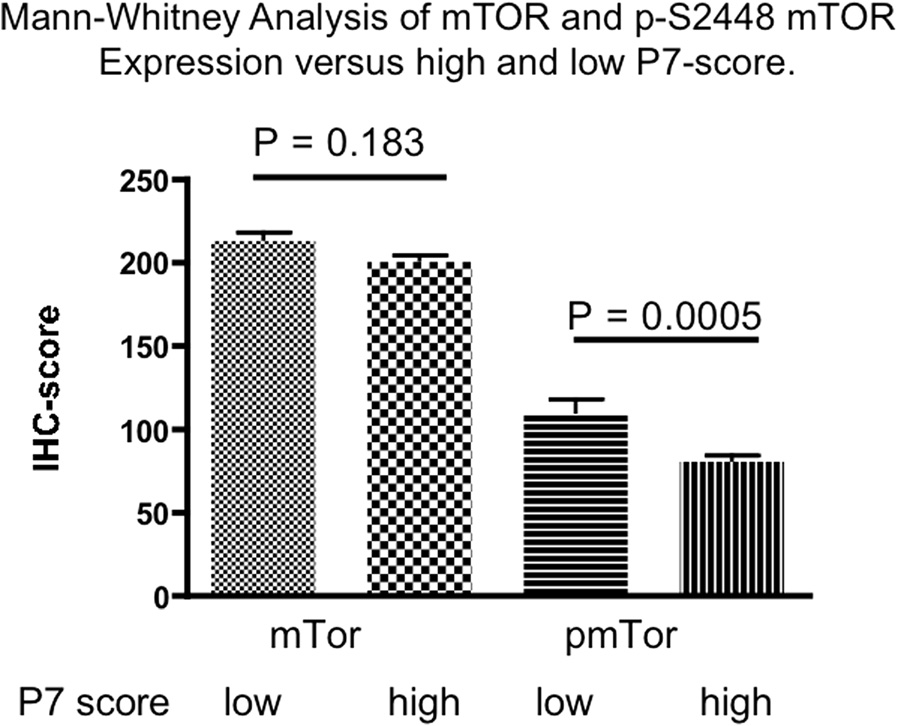
**p-S2448-mTOR expression as determined by immunohistochemistry is inversely related to P7-score in ER + primary breast cancer.** Tumors were dichotomized into high P7-score (>3) and low P7-score (<3). The histograms show mean ± SEM for the two groups. The median H -scores were significantly different between the two groups for p-S2448-mTOR but not total mTOR using a Mann–Whitney two tailed statistical analysis. ER, estrogen receptor; mTOR, mechanistic target of rapamycin; p-S2448-mTOR, mTOR phosphorylated on serine 2448; SEM, standard error of the mean.

Previously, we had determined expression of seven different phosphorylated residues on ERα in these same breast cancer samples from patients who subsequently received adjuvant tamoxifen therapy, and found that multiple tumors expressed combinations of phosphorylated ERα epitopes [5,6]. We also established that some phosphorylation sites were significantly associated with good and others with poor clinical outcome. From this, we defined an ERα phosphorylation score, taking into account the phosphorylation status of ERα at each of the seven sites interrogated. This so-called P7-score was significantly associated with OS from breast cancer death and RFS in multivariate analysis [6]. Due to the relationship of p-S2448 mTOR with clinical outcome in this cohort, we investigated the relationship of p-S2448 mTOR to different phosphorylated forms of ERα in these samples. p-S2448-mTOR was positively correlated with p-S118-ERα (r = 0.268, n = 308, *P* <0.0001), p-S167-ERα (r = 0.205, n = 325, *P* = 0.0002) and p-S282-ERα (r = 0.188, n = 304, *P* = 0.001), but negatively correlated with p-T311-ERα (r = −0.125, n = 307, *P* = 0.028). Previously [6] we had shown that detection of several phosphorylation sites on ERα, p-S104/106-ERα, p-S118-ERα, p-S167-ERα, p-S282-ERα and p-S294-ERα, was associated with a good clinical outcome while p-T311-ERα and p-S559-ERα were associated with poor clinical outcome. In the current study we found that p-mTOR was positively associated with p-S118-ERα, p-S167-ERα and p-S282-ERα but negatively associated with p-T311-ERα, which suggested an inverse relationship with the P7-ERα score and, indeed, p-S2448 mTOR expression was found to be negatively correlated with P7-ERα score (r = −0.23, n = 284, *P* <0.0001). When tumors were dichotomized into high P7-ERα score (defined by the clinically relevant ≥3 cut-off described previously) versus low P7-ERα score (<3) the IHC scores for p-S2448-mTOR were significantly higher in low versus high P7-ERα score tumors (median IHC score 90 versus 70, respectively, Mann–Whitney two-tailed *P* = 0.0005, Figure 2). These data further support an association of high p-S2448-mTOR with good prognosis. Since the P7-score remained significant on multivariate analysis as previously described (see Tables 1 and 2) but p-S2448-mTOR did not, the relationship of p-S2448-mTOR to the P7-score is likely driving its association with clinical outcome.

Nuclear staining for p-S2448-mTOR has been previously reported [20] and in the current cohort was detected in approximately 10% (38/352) of assessable cases. Nuclear p-S2448-mTOR was correlated with cytoplasmic p-mTOR (r = 0.495, n = 38, *P* = 0.0018) and while nuclear p-S2448-mTOR showed similar trends in terms of relationships to phosphorylated P7 ERα score, it was not analyzed further due to the small numbers of positive cases.

### Relationship of p70S6K to activated mTOR and phosphorylated ERα

To explore further the relationship of phosphorylated ERα to the activated mTOR pathway, specifically the mTOR complex 1 (mTORC1), TMA sections from the above breast cancer cohort were examined for the expression of p70S6K, a downstream target of p-S2448-mTOR within the mTORC1 [21]. Both nuclear and cytoplasmic staining for p-T389-p70S6K and total p70S6K has been reported and both were scored separately in the above cohort [22]. The majority of cases were positive for both cytoplasmic and nuclear p-T389-p70S6K as well as total p70S6K. As expected both cytoplasmic and nuclear p-T389-p70S6K and total p70S6K were positively correlated with both total and p-S2448-mTOR. Neither cytoplasmic nor nuclear p-T389-p70S6K was associated with the P7-ERα score. However, total nuclear p70S6K showed a weak inverse correlation with the P7-ERα score (r = − 0.183, n = 273, *P* = 0.0024). When tumors were dichotomized into high P7-ERα score (defined by the clinically relevant ≥3 cut-off described previously, [6]) versus low P7-ERα score (<3) the median IHC scores for total nuclear p70S6K were significantly higher in low versus high P7-ERα score tumors (median IHC score 62 versus 20, respectively, Mann–Whitney two tailed *P* = 0.034, Additional file 5: Figure S5). Positive correlations of total nuclear p70S6K expression with p-S104/106-ERα (r = 0.186, n = 249, *P* = 0.0033), p-S118-ERα (r = 0.175, n = 298, *P* = 0.0025), p-S167-ERα (r = 0.12, n = 315, *P* = 0.03) and p-S282-ERα (r = 0.128, n = 302, *P* = 0.026) were found. When tumors were dichotomized into high (defined by the median IHC ≥20) versus low total nuclear p70S6K expression, the median IHC for p-S104/106-ERα, p-S118-ERα, p-S167-ERα and p-S282-ERα were significantly higher in the high versus the low total nuclear p70S6K groups (Mann–Whitney two-tailed *P* = 0.0079; *P* = 0.0135; *P* = 0.02; *P* = 0.04, respectively). However, no associations of p70S6K (phosphorylated or total) with clinical outcome were found.

### Estrogen induces activation of mTOR and its downstream target p70S6K

**Figure 3:**
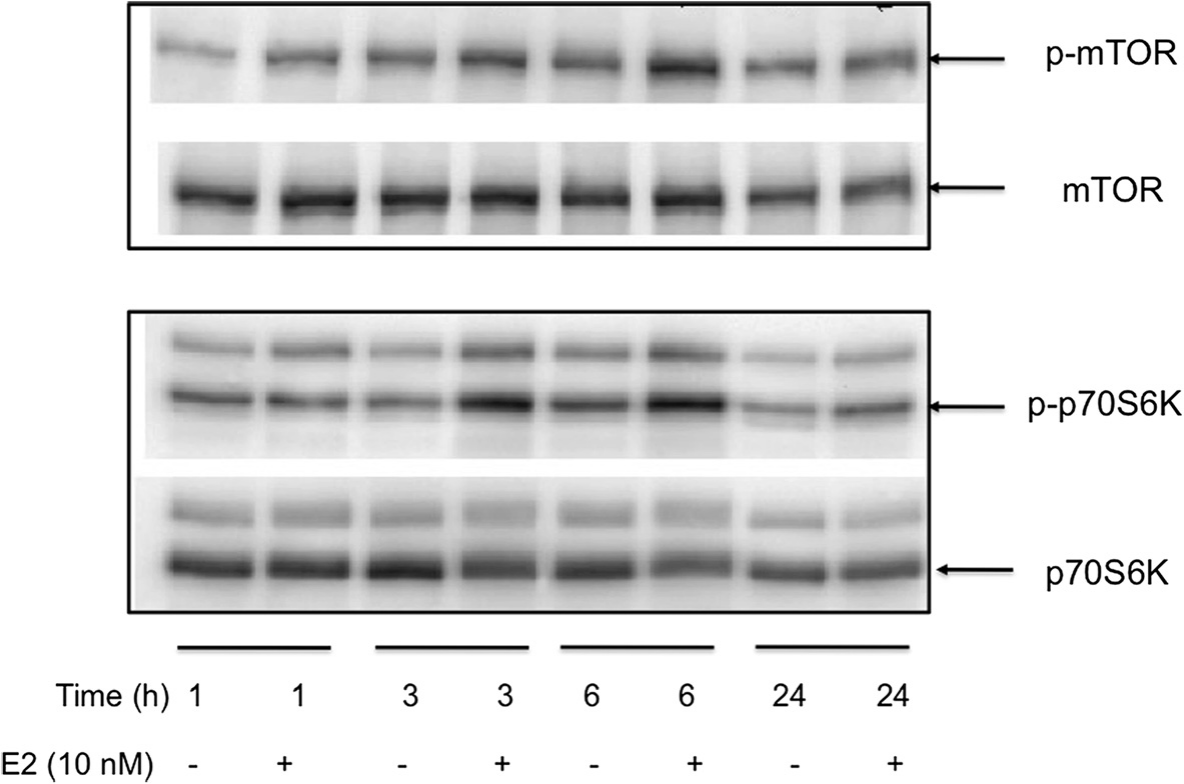
**Effect of estrogen treatment on p-S2448-mTOR and p-p70S6Kinase in MCF7 human breast cancer cells.** MCF7 were serum starved and estrogen depleted and then treated with and without E2 (10 nM) for the indicated time periods. Cell extracts were prepared and analyzed as described in the Methods section: 100 ug of extr0061ct was subject to Western blotting for p-mTOR, total mTOR, p-p70S6K and total p70S6K. Results represent one of four to seven independent experiments. Upper panel: for p-S2448-mTOR, E2 treatment for three hours showed a 1.5 ± 0.2 fold, n = 7 (mean ± SD) and at six hours 1.4 ± 0.3 fold, n = 5 (one way ANOVA *P* = 0.0006) increase. Middle panel: for p-T389-p70S6K, E2 treatment for three hours showed a 1.9 ± 0.5 fold, n = 10 (mean ± SD) and at six hours 1.6 ± 0.3 fold, n = 10 (one way ANOVA *P* < 0.0001) increase. ANOVA, analysis of variance; E2, estradiol; mTOR, mechanistic target of rapamycin; p-S2448 mTOR, mTOR phosphorylated on serine 2448; SD, standard deviation.

Several studies have indicated that estrogen can induce activation of the mTOR pathway in estrogen target tissues including breast cancer cells [23-26]. Activation of the mTOR pathway was usually established by demonstrating activation or inhibition of up- or down-stream targets of mTOR [13,27]. However, the ability of estrogen to regulate phosphorylation of mTOR in breast cancer cells has not been reported. The observed correlation between a direct marker of mTOR activation, p-S2448 mTOR, and the P7-ERα score in ERα + primary breast cancer cases prompted us to investigate the ability of estrogen to regulate phosphorylation of mTOR in MCF7 human breast cancer cells. MCF7 cells were depleted of estrogen and serum starved overnight prior to treatment with estrogen for various times. As shown in Figure 3, estradiol (E2) treatment for three to six hours was associated with a small (mean ± SD; at three hours 1.5 ± 0.2 fold, n = 7; at six hours 1.4 ± 0.3 fold, n = 5; one way analysis of variance (ANOVA) *P* = 0.0006) but consistent induction of p-S2448 mTOR. Furthermore, E2 treatment for three and six hours also resulted in the phosphorylation of p70S6K at threonine 389 (Figure 3) (mean ± SD; for three hours 1.9 ± 0.5 fold n = 10; at six hours 1.6 ± 0.3 fold, n = 10, one way ANOVA *P* <0.0001). These latter results are in agreement with previous reports [27]. These data provide support for the ability of estrogen to affect activation of mTOR and one of its downstream targets in MCF7 human breast cancer cells.

**Figure 4:**
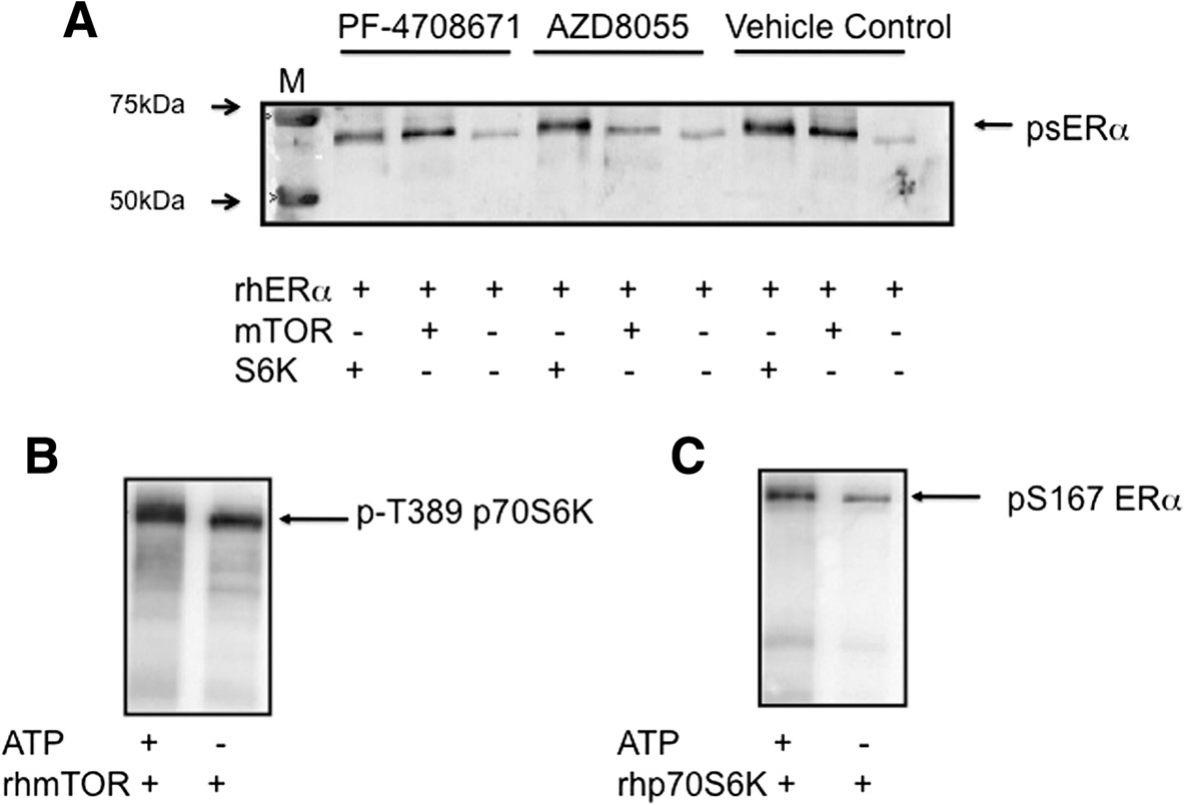
**mTOR can phosphorylate ERα *in vitro*. A)** 300 ng of rh-ERα alone (full-length human ERα), or in the presence of 100 ng of mTOR (mTOR/Raptor/MLST8 complex, catalytic domain) or 100 ng of p70S6K was subjected to *in vitro* kinase assays in the presence of 0.2 mM ATP for 30 minutess at 30°C. Reactions were pre-incubated for 15 minutess with DMSO (vehicle control) or with selective kinase inhibitors (100 nM mTOR inhibitor AZ8055 or 10 μM p70S6K inhibitor PF-4708671). Following incubation, an aliquot of the reaction mix was subjected to Western blotting and visualized with an antibody specific for phospho-serine residues. **B)** 100 ng of recombinant human p70S6K was incubated with 100 ng of rh-mTOR (catalytic domain) with and without ATP as described in the Methods section. At the end of the incubation an aliquot of each reaction was subjected to Western blotting and visualized with an antibody specific for p-T389-p70S6K. **C)** 300 ng of rh-ERα and 100 ng of recombinant full-length p70S6K were incubated together in the presence or absence of ATP as described in the Methods section. At the end of the incubation an aliquot of each reaction was subjected to Western blotting and visualized with an antibody specific for pS167-ERα. M = molecular mass marker. DMSO, dimethyl sulfoxide; E2, estradiol; mTOR, mechanistic target of rapamycin; rh, recombinant human.

**Figure 5:**
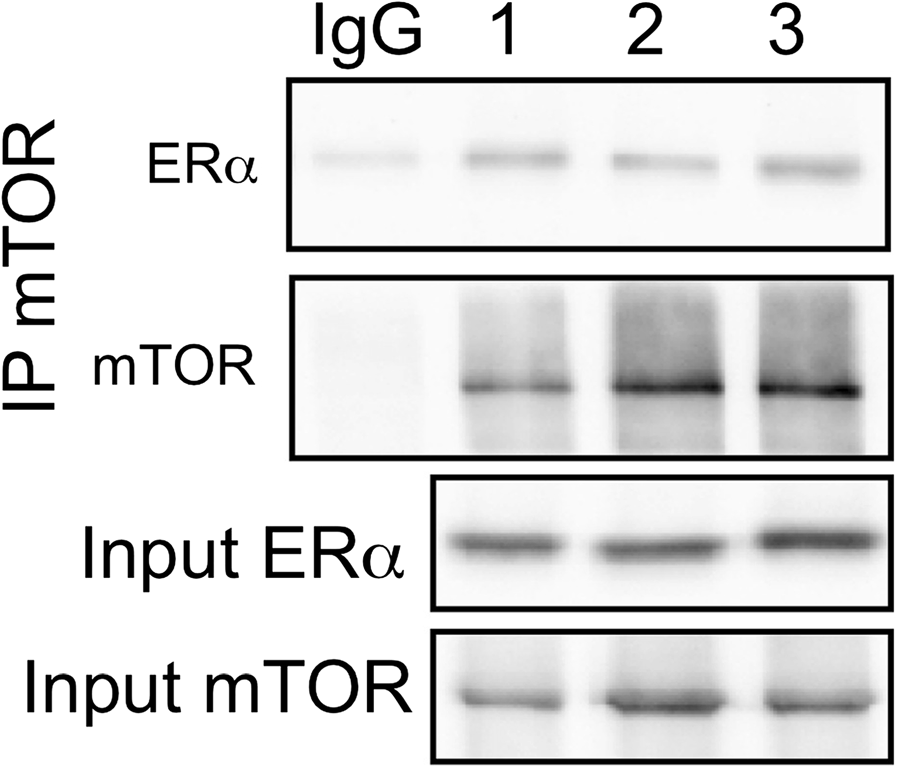
**ERα is co-immunoprecipitated with mTOR from MCF7 cells.** MCF7 cells were grown in 5% CM (lane 1) or MCF7 were serum starved and estrogen depleted and then treated with (lane 3) and without E2 (lane 2) for 60 minutess. Cells were harvested, crosslinked with DSP and immunoprecipitated with an isotype-matched but irrelevant antibody (IgG) or an antibody specific for mTOR (sc-1549-R). Aliquots of immunoprecipitated complexes were subjected to Western blot analysis with antibodies to ERα or mTOR, as shown. Levels of ERα and mTOR in input lysates are shown in the two lower panels. A representative experiment is shown from a total of three independent experiments. CM, complete medium; DSP, dithiobis(succinimidylpropionate); E2, estradiol; ERα, estrogen receptor α; IgG, immunoglobulin G; mTOR, mechanistic target of rapamycin,

Previously, we found that several serine residues in ERα resided within motifs that suggested their potential to be FRAP/mTOR substrates [28]. Therefore, to determine the potential of mTOR to directly phosphorylate ERα, an *in vitro* kinase assay was performed using full-length recombinant human ERα (rh-ERα) incubated with the catalytic domain of recombinant human mTOR (rh-mTOR) or with full-length recombinant human-p70S6K (rh-p70S6K), since it has previously been shown to phosphorylate ERα. As expected, rh-p70S6K increased the phosphorylation of rh-ERα at serine residues by six fold (mean, range three to nine, n = 2) and importantly rh-mTOR increased the phosphorylation of rh-ERα at serine residues by 4.4 ± 1.7 fold (mean ± SD, n = 3) (Figure 4A). Preincubation with a selective mTOR inhibitor, AZD 8055, inhibited the serine phosphorylation of rh-ERα in the presence of mTOR but not p70S6K, and preincubation with a selective inhibitor to p70S6K, PF-4708671, inhibited serine phosphorylation of rh-ERα in the presence of p70S6K but not mTOR (Figure 4A). As a positive control for the activity of rh-mTOR, an aliquot was incubated with rh-p70S6K plus or minus ATP and western blotted for p-T389-p70S6K (Figure 4B). As expected, rh-mTOR increased the phosphorylation of T389-p70S6K. Another control, where rh-ERα was incubated with rh-p70S6K with and without ATP and western blotted for p-S167-ERα also showed the expected increase in p-S167-ERα (Figure 4C). These data establish at least the potential for mTOR to phosphorylate ERα.To determine if mTOR and ERα could interact in intact cells, appropriately treated cells were cross-linked using DSP and co-immunoprecipitation (co-IP) undertaken. Figure 5 shows that ERα was co-immunoprecipitated with antibodies specific for mTOR but not with irrelevant antibodies, although little difference due to treatment was detected. These data suggest that ERα can exist in a complex with mTOR supporting a possible direct interaction and regulation of the two proteins. Similar trends of interaction were obtained when antibodies to p-mTOR were used for co-IP (data not shown).

## Discussion

Since the mTOR pathway is a target for inhibition in cancer treatment, and some previous studies have reported a positive association of high levels of pS2448-mTOR with poor prognosis in breast cancer [21,29-32], the relationship we have found between p-S2448 mTOR and the P7-score reflecting good outcome in patients subsequently treated with adjuvant tamoxifen therapy, was unexpected. However, the breast cancer cohort examined in the current study is distinct from previously published cohorts, as it consisted entirely of primary ERα+, sporadic cases, which contained both node positive and negative cases, the majority of women were postmenopausal and the patients all received adjuvant tamoxifen treatment following surgery and radiation. Also, due to the nature of the tumor collection at the MBTB, the cases are biased to larger sized tumors [15,16]. Previously reported studies included cohorts which: were ERα negative with the majority being triple negative breast cancers [21], contained both ERα + and ERα- cases with the majority being defined as low risk (small tumor size and node negative) [32], had no information available concerning therapies [30], consisted of mainly familial breast cancer cases where few are ERα + [29] or consisted of a cohort in which only 50% of tumors were ERα+, or more than 60% of the women were under 50 years of age [31].

Our data show that high levels of p-S2448 mTOR expression are associated with good clinical outcome in ERα + patients, subsequently treated with tamoxifen, in univariate but not multivariate analysis. We also found p-S2448 mTOR expression was inversely associated with the P7-ERα score, which is a prognostic factor (that is, significantly associated with outcome on multivariate analysis) in tamoxifen treated patients. This suggests that activation of mTOR in this tumor cohort is associated with an intact estrogen (ligand) dependent signaling pathway [33]. It is well known that if growth and survival of a tumor depends on estrogen and, therefore, an intact, functional estrogen-dependent signaling pathway, then endocrine therapies such as tamoxifen and aromatase inhibitors are most likely to be of benefit to the breast cancer patient [34]. Since the mTOR pathway is a central regulator of cell growth, metabolism and survival [11], it makes sense that estrogen (through ERα) would regulate the mTOR pathway in cells that are dependent on estrogen for growth, metabolism and survival. On the other hand, activation of mTOR by amplification of growth factor receptor pathways would be expected to short circuit estrogen dependent regulation and be associated with resistance to endocrine therapy [35]. This is supported by experimental models as well as clinical associations and, as such, provides the rationale for combining endocrine therapies and mTOR inhibitors, such as rapamycin [11].

Clinical data supporting regulation of the mTOR pathway by an intact estrogen signaling pathway are derived from a neoadjuvant trial of letrozole, an aromatase inhibitor, where decreased detection of p-S2448-mTOR following letrozole treatment was associated with a significantly longer disease-free survival [25]. This latter study suggests that in some breast cancers estrogen is regulating the activation of mTOR and removal of estrogen, through aromatase inhibition, decreased mTOR activation (at least as measured by decreased p-S2448-mTOR). Our current data are, therefore, consistent with this latter study.

The phosphorylated form of mTOR, assessed in the current study, p-S2448, is a measure of activated mTORC1 [21]. Therefore, activated mTORC1 was found inversely related to the phosphorylation code or P7-score of ERα in this breast cancer cohort [6]. While a low P7-ERα score represents more phosphorylation at ERα sites associated with good prognosis (for example, S118, S167, S282) and clinical outcome, a high P7-ERα score represents more phosphorylation at ERα sites associated with poor prognosis (for example, T311) and clinical outcome in patients subsequently treated with tamoxifen [6]. Therefore, the inverse relationship of activated mTORC1 with P7-ERα score is consistent with activated mTORC1 being associated with better clinical outcome. However, since the P7-score accounts for most of the variation that can also be ascribed to p-mTOR, and they are correlated, not surprisingly, this contributes to a loss of significance of p-mTOR when they appear together in a multi-predictor model.

An exciting possibility supported by our data, is that kinases associated with the activated mTORC1, including mTOR itself or kinases regulated by mTORC1, could be involved in phosphorylation of the ERα. High prediction scores were found using the Kinexus Phosphonet kinase predictor [36] for Ser118 and Ser294 in ERα as substrates for mTOR. P70S6K is a downstream target of the mTORC1 pathway [37] and has been shown to phosphorylate ERα on Ser167 in cells in culture [14]. However, we found no relationship of p-T389-p70S6K expression with p-S167-ERα or P7-score in the current cohort. A weak correlation between total nuclear p70S6K and p-Ser167, as well as other phosphorylated ERα sites associated with good prognosis was observed, which translated into a weak inverse correlation with the P7-score. In contrast to p-S2448-mTOR, no relationship to clinical outcome was found for total or phosphorylated p70S6K, nuclear or cytoplasmic.

Previous reports have suggested that estrogen can regulate the mTOR pathway. Activation of mTOR was most often determined by demonstrating activation of p70S6K, a downstream target of mTOR, although other markers of mTOR activation have also been used [23,24,27]. However, direct evidence of mTOR phosphorylation was missing. We show here for the first time, as far as we know, that phosphorylation of mTOR at serine 2448 can be induced by estrogen in a time dependent manner in MCF7 breast cancer cells and mTOR can be coimmunoprecipitated with ERα in these cells. These are small but reproducible effects. A possible reason for the small effect may be that MCF7 cells are cells derived from a pleural effusion, that is, metastatic breast cancer, and the cells are usually only estrogen responsive and not estrogen dependent for growth and survival, in cell culture [38,39]. Therefore, they may not be an exact model for estrogen dependent ER + primary breast cancer *in vivo*, as also suggested by a recent publication [40]. We also demonstrate that mTOR is capable of phosphorylating ERα *in vitro* further supporting a relationship between ER activation and mTOR activity. Our *in vitro* and *in vivo* data suggest that there may be multiple ways in which the ER pathway crosstalks with the mTOR pathway, with both feed forward and feedback interactions such that when the balance is perturbed resistance to endocrine therapies can develop. Such interactions and their regulation require further investigation.

Preanalytical variables around tissue collection are now recognized to be important and a source of variation, particularly associated with PTMs, such as phosphorylation. PTMs are dynamic and marked changes in phosphorylated epitopes that can occur in samples due to the type of surgery, the type of biopsy and fixation time, and other factors that may result in erroneous conclusions [41-43]. Although we cannot completely exclude effects due to such issues, there are a number of reasons why we feel issues of tissue collection and differential fixation are unlikely to explain the results we have obtained. Firstly, the MBTB is populated primarily with samples that were left over from tissue collected for ER/PR assays performed by ligand binding. While some study samples were from lumpectomies and some are from mastectomies during the era that the samples were collected (1988 to 2000), tumors were mostly large and palpable clinically and on gross dissection and handled the same way (that is, rapidly assessed and sampled because of the priority given to fresh sampling to conduct ligand binding assays as this was the provincial standard at the time for all breast cancer cases. Therefore, our MBTB samples were derived in all cases from a pathology resection specimen that at the time of surgery was rapidly assessed by the pathologist and both mastectomies and lumpectomies were dissected and sampled immediately (mean time approximately 50 minutess) to obtain a fresh tumor sample that was then frozen in the pathology laboratory. The samples were then transferred to the provincial laboratory and frozen fragments cut from each on a chilled surface for the clinical ER/PR ligand binding assay. After the assay was completed and reported, the remaining frozen samples were passed to the MBTB where all samples are divided on a dry ice chilled surface to create mirror image blocks of tissue and both blocks are returned to the freezer. Then a block (typically 3 mm × 5 mm × 10 mm size) of each pair is removed and immersed in formalin and fixed for a set period (24 hours) and then processed in consistent batches [15,44,45]. Therefore, the variation in delay in sampling and freezing of tissues initially is small and the delay and variation in fixation is also minimal and all blocks are small and fixed for a standard length of time.

Secondly, although Pinhel *et al.,*[41] show that levels of phospho-epitopes are consistently lower in specimens where there was a delay in fixation, there is still a very good correlation between cores and resection levels in individual cases. Therefore, mixing cores and resection specimens in a single study is likely to suffer from issues of variability between types of specimens but still their data suggest that the relative expression levels within each type of specimen are maintained. Therefore, while resection specimens have lower levels of expression than cores this would mostly affect the linear range of detection rather than the overall rank order among cases. Our study examines one type of biospecimen (resections) and all were collected in a relatively standardized fashion. Furthermore, our timed collection data suggest that there was minimal loss of epitope in our cohort.

Thirdly, we show a relative and inverse correlation of p-mTOR with overall P7-ERα score, which is a complex relationship (negative and positive) of detection of several different p-ERα sites some of which we have shown are related to good and some to poor clinical outcome [6]. Despite the possibility that different phospho-epitopes are potentially differentially influenced by fixation time, we have identified a biologically plausible relationship. The hypothesis derived from this is also consistent with some results in the literature (i.e. correlative in neoadjuvant trials [25]; and those using laboratory models both cell lines and target tissues from animals [24,26,27]). Furthermore, when we show data using laboratory models which have tested the hypothesis with some success. This also suggests that issues of fixation are less likely an issue.

## Conclusions

In summary, in primary tumors from an ER + cohort of breast cancer patients who were subsequently treated with tamoxifen, increased activated mTORC1 was found to be associated with better clinical outcome but was not an independent prognostic factor. Since activated mTORC1 was also inversely correlated with the phosphorylation score of ERα (P7-score), and the P7-score has previously been shown to be a significant independent prognostic factor in this cohort, we conclude that activated mTORC1 is due to an intact estrogen dependent signaling pathway in this breast cancer cohort.

## Abbreviations

Akt: serine-threonine protein kinase encoded by the v-akt murine thymoma viral oncogene homolog gene; CM: complete medium; (D)MEM: (Dulbecco’s) modified eagles medium; E2: estradiol; ERα: estrogen receptor-alpha; FBS: fetal bovine serum; FRAP: FKBP12-rapamycin complex-associated protein; HR: hazard ratio; IHC: immunohistochemistry; IP: immunoprecipitation; MBTB: Manitoba Breast Tumor Bank; mTOR: mechanistic target of rapamycin; mTORC1: mechanistic target of rapamycin complex 1; OS: overall survival; p70S6K: p70 S6 kinase; P7-score: a measure of the presence of up to seven phosphorylated ERα epitopes by immunohistochemistry in breast cancer biopsy samples; PI3K: phosphatidylinositol-4,5-bisphosphate 3-kinase; PBS: phosphate-buffered saline; PR: progesterone receptor; p-S2448 mTOR: mTOR phosphorylated on serine 2448; p-T389 p70S6K: p70S6K phosphorylated on threonine 389; PTM: post-translational modifications; RFS: relapse free survival; TMA: tissue microarrays.

## Competing interests

The authors declare that they have no competing interests.

## Authors’ contributions

AS contributed to the project design, conception, acquisition of data, analysis and interpretation of data, drafting and revising the manuscript; MCB contributed to acquisition of data, analysis and interpretation of data, drafting and revising the manuscript; DJ, contributed to acquisition of new data, analysis of data and revising the manuscript; TB, contributed to acquisition of data, analysis of data, and revising the manuscript; SS, contributed to project design, acquisition of data, analysis of data, and revising the manuscript; CP, contributed to acquisition of data, analysis and interpretation of data, and revising the manuscript; ZN, contributed to project design, analysis and interpretation of data, drafting and revising the manuscript; PHW and LCM contributed to the project design, conception, analysis and interpretation of data, drafting and revising the manuscript and overall coordination and supervision of the study. All authors participated in writing, editing and final approval of the manuscript. All authors read and approved the final manuscript.

## Supplementary Material

Additional file 1: Figure S1 Immunohistochemical validation of P-S2448-mTOR antibodies in biopsies of representative human invasive breast cancers cores represented on TMAs. IHC was performed as described in the Materials and Methods. Adjacent sections of cores from breast cancer cases represented on a test TMA available in MBTB, where **(A)** stained with the p-S2448-mTOR antibody alone showing cytoplasmic staining; **(B)** an adjacent section of the cores using p-S2448-mTOR antibody antibody pre-absorbed with excess of the p-S2448-mTOR phosphorylated peptide; or **(C)** pre-absorbed with excess irrelevant ERα peptide phosphorylated at S118; or **(D)** pre-absorbed with the non-phosphorylated mTOR peptide; **(E)** stained with phospho-p70S6K (p-p70S6K) antibody alone; **(F)** an adjacent section of the cores using phospho-p70S6K antibody pre-absorbed with excess of the phosphorylated p70S6K peptide used to raise the antibody. All magnifications x 100. Click here for file

Additional file 2: Figure S2 Investigation of the expression of p-S2448-mTOR and p-T389-p70S6K in breast tumors *in vivo* due to tissue collection time. The timed collection cohort of tumors was also divided into groups based on collection times of ≤30 minutess versus >30 minutess. Mann–Whitney two-tailed analyses showed no significant differences in the IHC score for either phospho-epitope between the two time groups. Click here for file

Additional file 3: Figure S3 mTOR and p-S2448-mTOR expression as determined by immunohistochemistry is inversely related to nodal status in ER + primary breast cancer. Tumors were divided into node negative and positive categories and the histograms show the means ± SEM of the two groups. The median IHC-scores for mTOR and p-mTOR were significantly different between node positive and negative subgroups using a Mann–Whitney two tailed analysis. Click here for file

Additional file 4: Figure S4 mTOR expression as determined by immunohistochemistry is inversely related to tumor size in ER + primary breast cancer. Tumors were dichotomized into small (<2 cm) and large (>2 cm) size. The histograms show mean ± SEM for the two groups. The median H-scores for mTOR were significantly different between the two groups for total mTOR but not p-S2448-mTOR using a Mann–Whitney two tailed statistical analysis. Click here for file

Additional file 5: Figure S5 Nuclear p70S6K expression as determined by immunohistochemistry is inversely related to P7 score in ER + primary. Tumors were dichotomized into high P7 score (>3) and low P7 score (<3). The histograms show mean ± SEM for the two groups. The median H-scores were significantly different between the two groups for total nuclear p70S6K expression using a Mann–Whitney two tailed statistical analysis. Click here for file
