# Supplementary figures and images for: The mechanistic target for rapamycin pathway is related to the phosphorylation score for estrogen receptor-α in human breast tumors in vivo
Source: Breast Cancer Res. 2014 May 22;16(3):R49. doi: 10.1186/bcr3660 (PMC4076628; doi:10.1186/bcr3660)

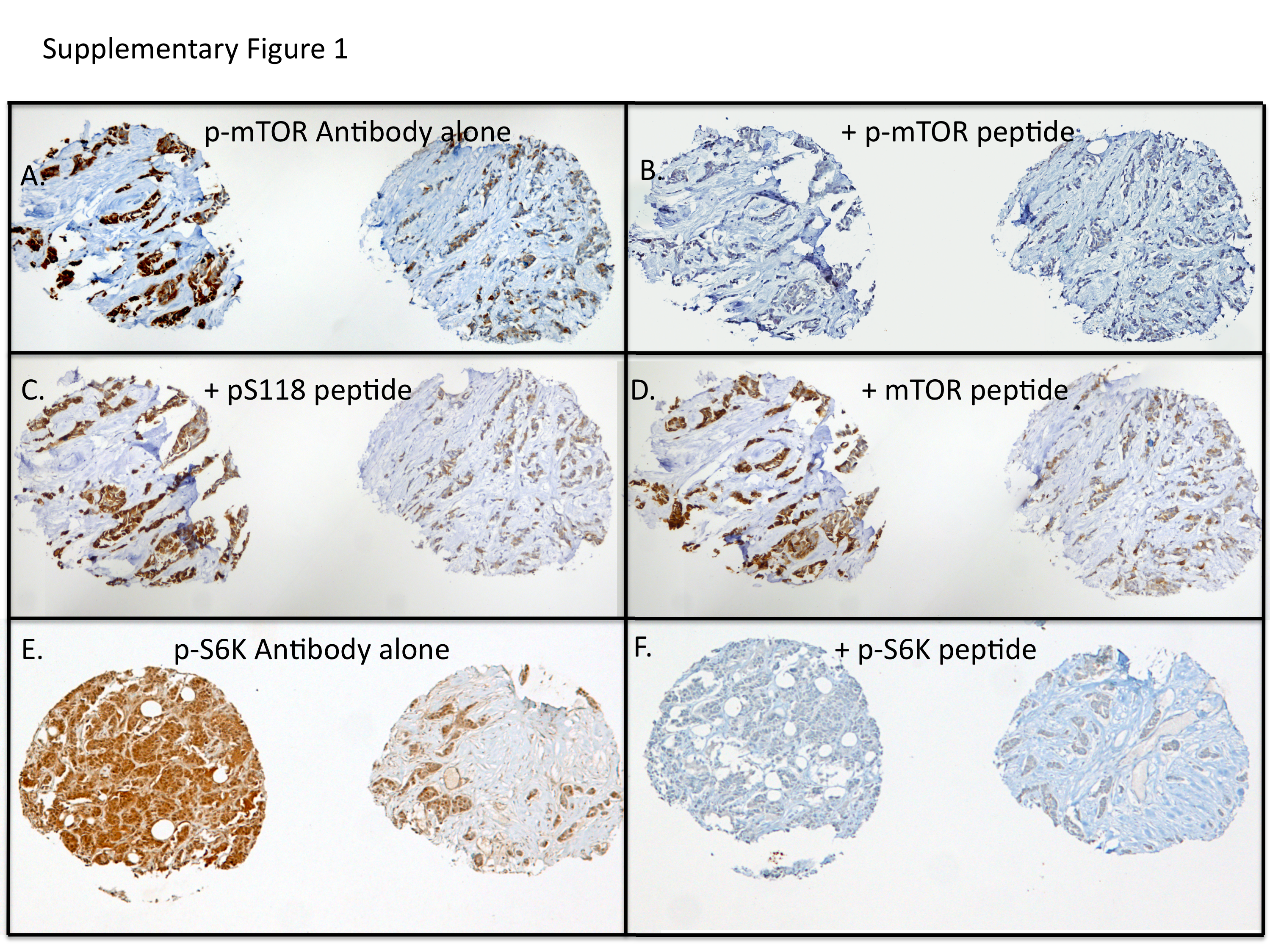

Supplement: Additional file 1: Figure S1 — Immunohistochemical validation of P-S2448-mTOR antibodies in biopsies of representative human invasive breast cancers cores represented on TMAs. IHC was performed as described in the Materials and Methods. Adjacent sections of cores from breast cancer cases represented on a test TMA available in MBTB, where (A) stained with the p-S2448-mTOR antibody alone showing cytoplasmic staining; (B) an adjacent section of the cores using p-S2448-mTOR antibody antibody pre-absorbed with excess of the p-S2448-mTOR phosphorylated peptide; or (C) pre-absorbed with excess irrelevant ERα peptide phosphorylated at S118; or (D) pre-absorbed with the non-phosphorylated mTOR peptide; (E) stained with phospho-p70S6K (p-p70S6K) antibody alone; (F) an adjacent section of the cores using phospho-p70S6K antibody pre-absorbed with excess of the phosphorylated p70S6K peptide used to raise the antibody. All magnifications x 100. [file bcr3660-S1.png]

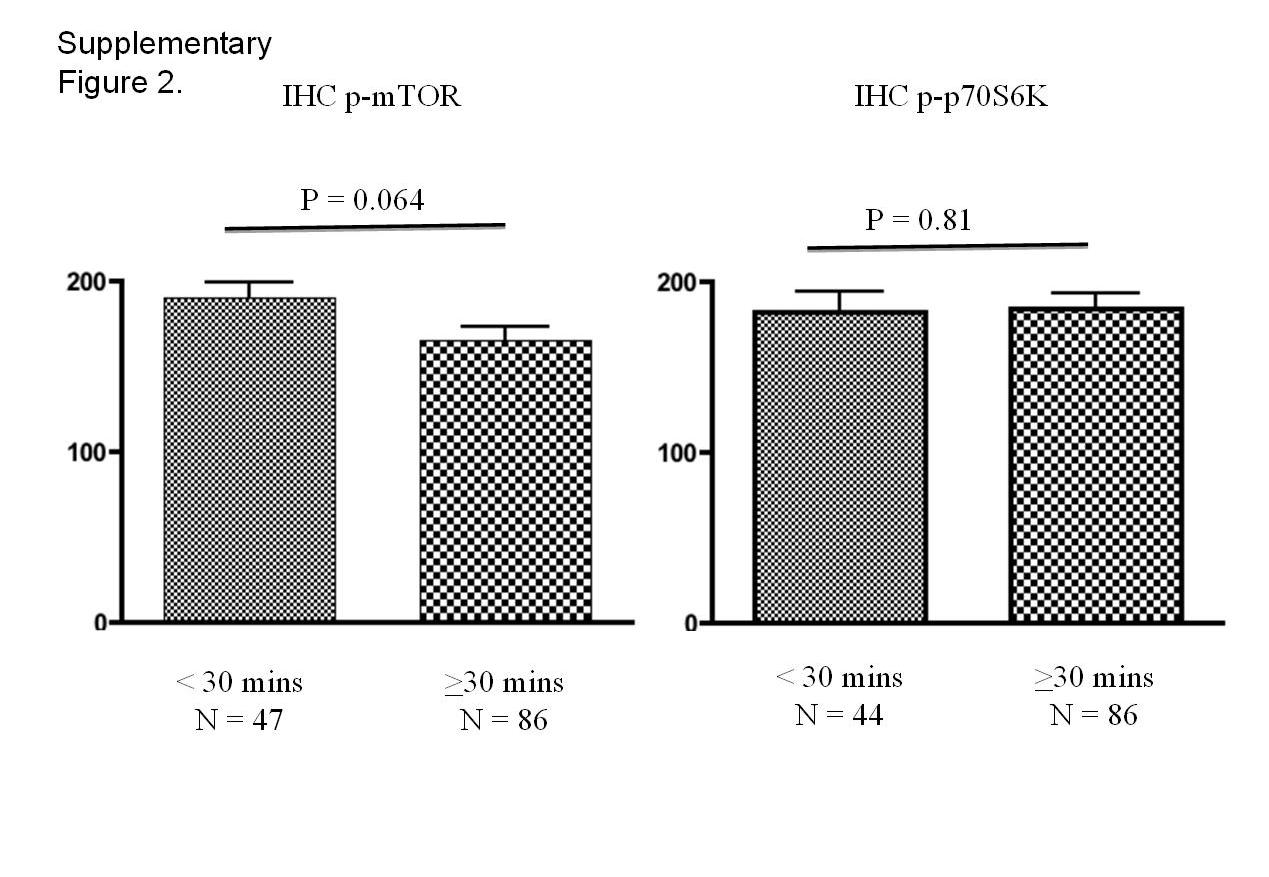

Supplement: Additional file 2: Figure S2 — Investigation of the expression of p-S2448-mTOR and p-T389-p70S6K in breast tumors in vivo due to tissue collection time. The timed collection cohort of tumors was also divided into groups based on collection times of ≤30 minutess versus >30 minutess. Mann–Whitney two-tailed analyses showed no significant differences in the IHC score for either phospho-epitope between the two time groups. [file bcr3660-S2.JPG]

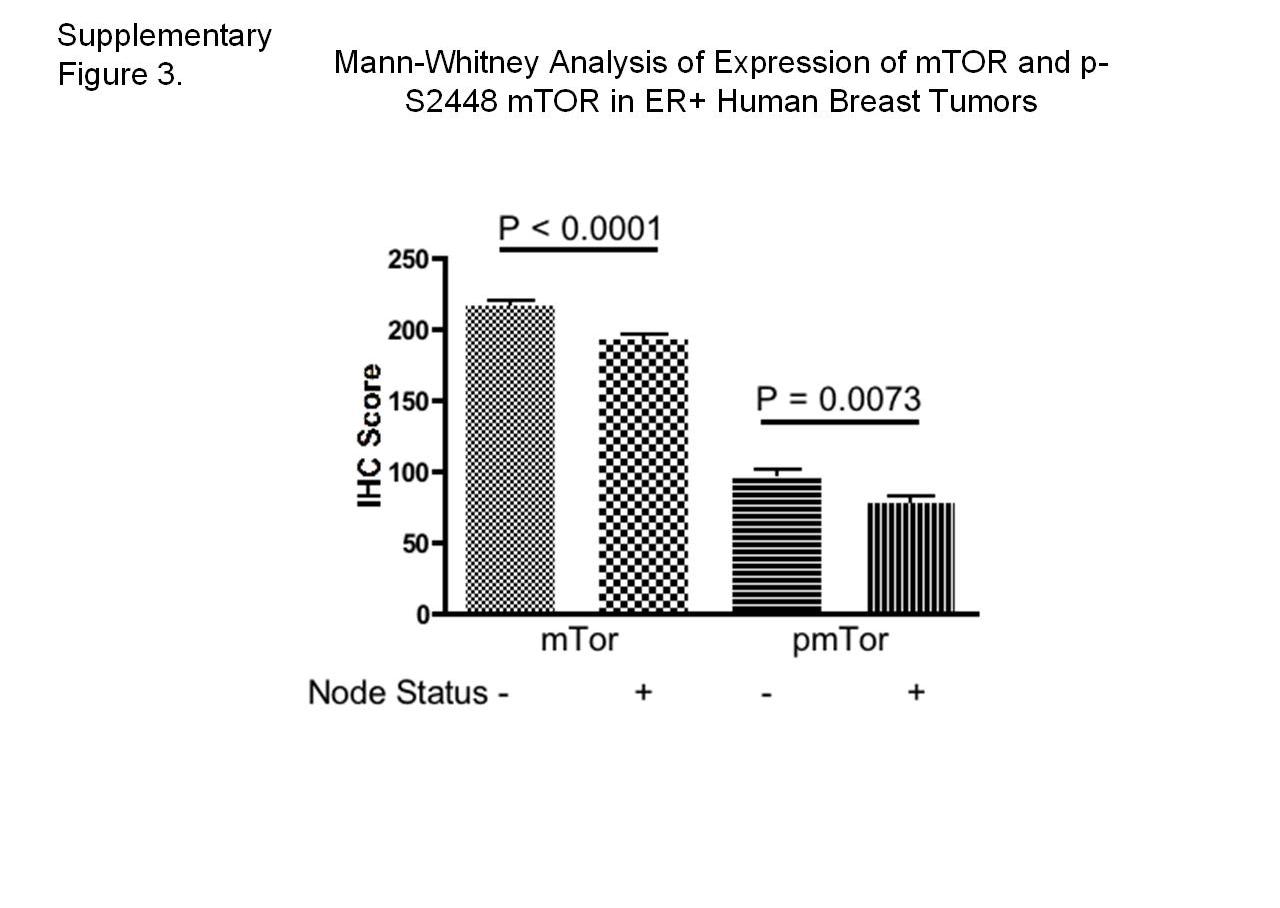

Supplement: Additional file 3: Figure S3 — mTOR and p-S2448-mTOR expression as determined by immunohistochemistry is inversely related to nodal status in ER + primary breast cancer. Tumors were divided into node negative and positive categories and the histograms show the means ± SEM of the two groups. The median IHC-scores for mTOR and p-mTOR were significantly different between node positive and negative subgroups using a Mann–Whitney two tailed analysis. [file bcr3660-S3.JPG]

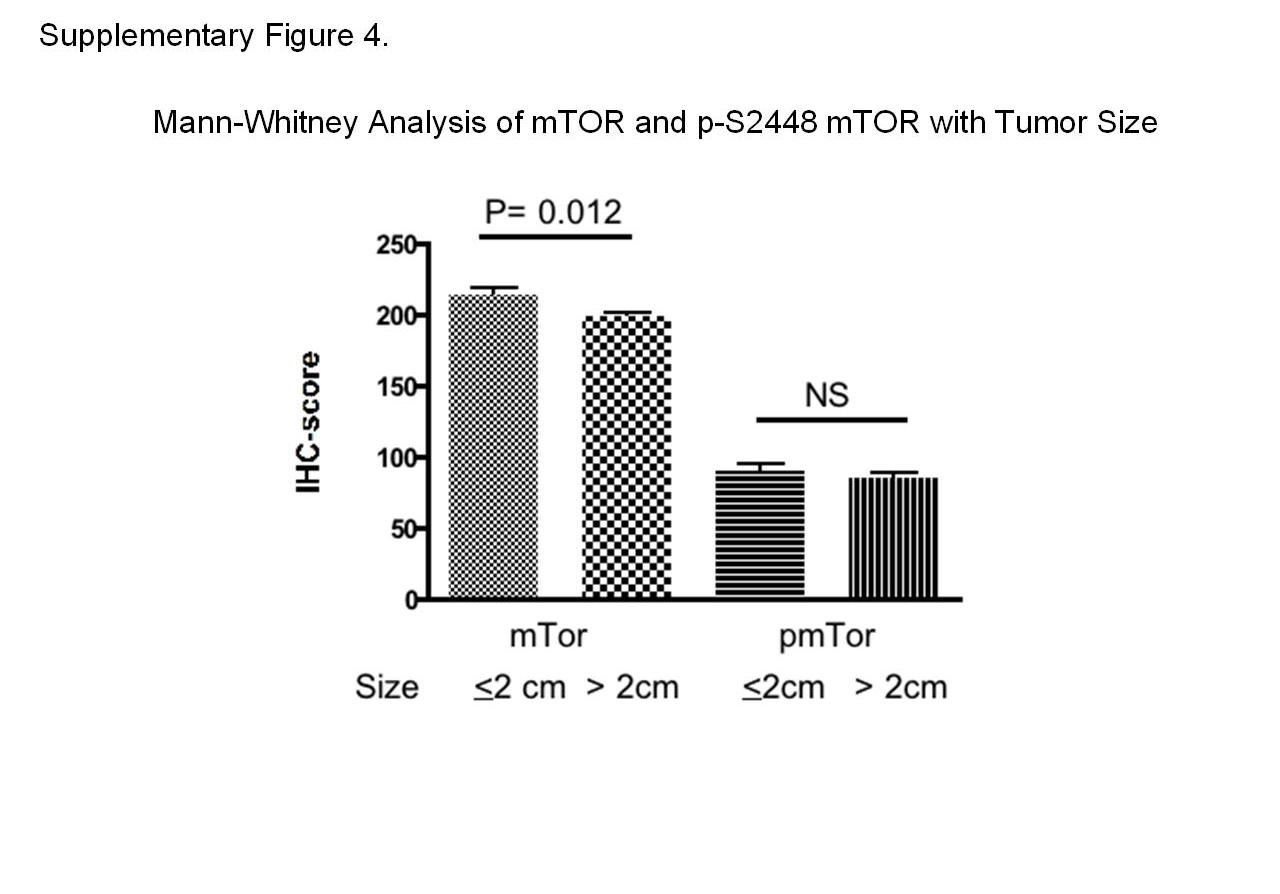

Supplement: Additional file 4: Figure S4 — mTOR expression as determined by immunohistochemistry is inversely related to tumor size in ER + primary breast cancer. Tumors were dichotomized into small (<2 cm) and large (>2 cm) size. The histograms show mean ± SEM for the two groups. The median H-scores for mTOR were significantly different between the two groups for total mTOR but not p-S2448-mTOR using a Mann–Whitney two tailed statistical analysis. [file bcr3660-S4.JPG]

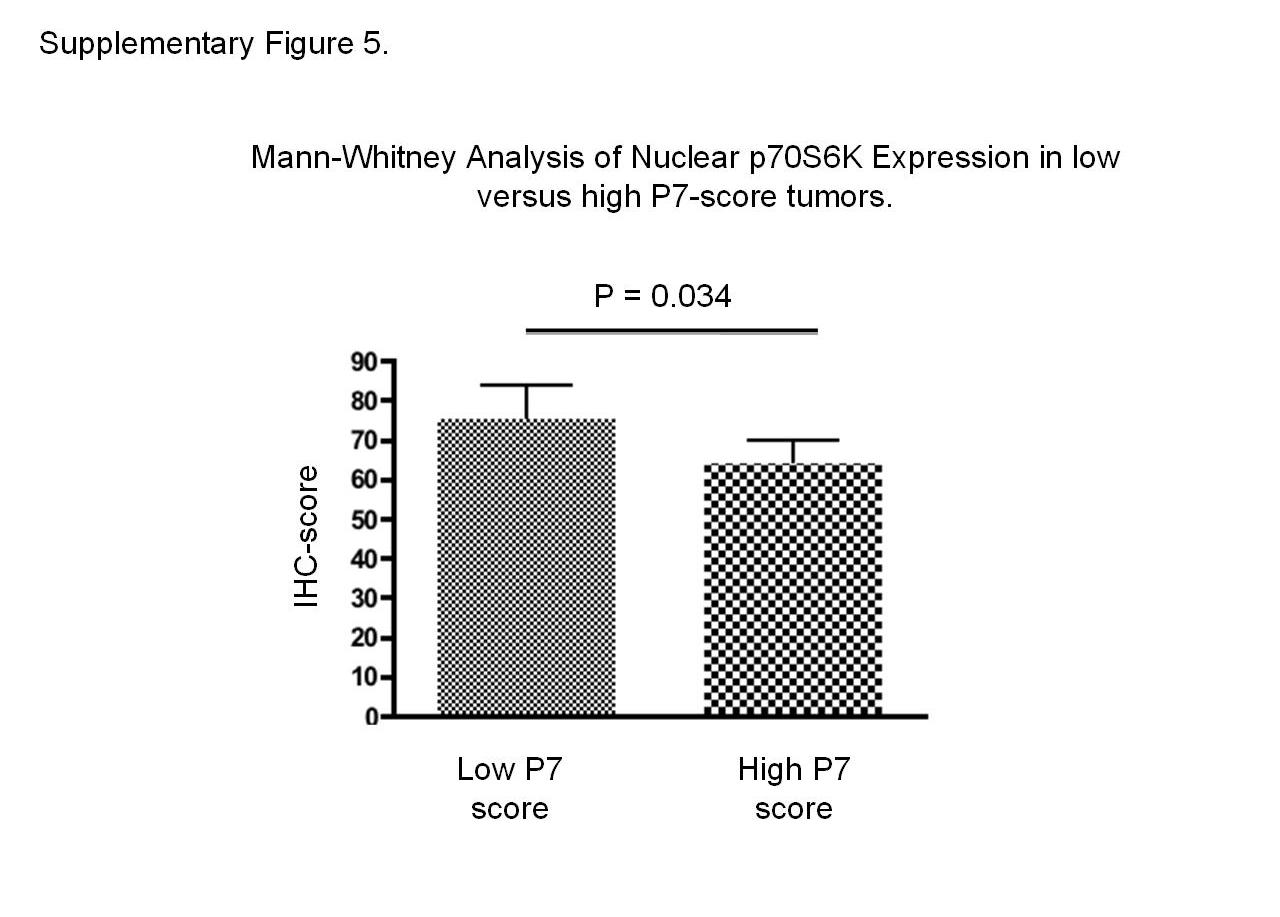

Supplement: Additional file 5: Figure S5 — Nuclear p70S6K expression as determined by immunohistochemistry is inversely related to P7 score in ER + primary. Tumors were dichotomized into high P7 score (>3) and low P7 score (<3). The histograms show mean ± SEM for the two groups. The median H-scores were significantly different between the two groups for total nuclear p70S6K expression using a Mann–Whitney two tailed statistical analysis. [file bcr3660-S5.JPG]
